# Population Genomic Scans for Natural Selection and Demography

**DOI:** 10.1146/annurev-genet-111523-102651

**Published:** 2024-11-14

**Authors:** Xiaoheng Cheng, Matthias Steinrücken

**Affiliations:** 1 Department of Ecology and Evolution, University of Chicago, Chicago, Illinois, USA; 2 Department of Human Genetics, University of Chicago, Chicago, Illinois, USA

**Keywords:** population genetics, genomic variation, natural selection, demographic history

## Abstract

Uncovering the fundamental processes that shape genomic variation in natural populations is a primary objective of population genetics. These processes include demographic effects such as past changes in effective population size or gene flow between structured populations. Furthermore, genomic variation is affected by selection on nonneutral genetic variants, for example, through the adaptation of beneficial alleles or balancing selection that maintains genetic variation. In this article, we discuss the characterization of these processes using population genetic models, and we review methods developed on the basis of these models to unravel the underlying processes from modern population genomic data sets. We briefly discuss the conditions in which these approaches can be used to infer demography or identify specific nonneutral genetic variants and cases in which caution is warranted. Moreover, we summarize the challenges of jointly inferring demography and selective processes that affect neutral variation genome-wide.

## 1. INTRODUCTION

A central goal of the field of population genetics is to unravel the processes that shape the genetic and phenotypic diversity observed in natural populations. Two fundamental processes underlying this diversity are selection on genetic variation, resulting from fitness differences between individuals due to genetic factors, and demographic processes, such as past changes in effective population size and population structure. Aside from answering fundamental biological questions, characterizing these processes has practical implications, for example, in conservation genetics (148) and in the interpretation of genomic variation in medical contexts (138). Because of the fundamental nature of these processes, many methods have been developed to characterize adaptive genetic variation (75, 98, 142) or demographic processes (88, 93, 134) through the use of population genomic data. Moreover, technological advances have enabled the collection of large population genomic data sets to which these methods have been applied in order to uncover the relationships between different population groups and characterize genetic variation under selection in different scenarios.

Population genetic methods are often applied to genomic data sets of single-nucleotide polymorphisms (SNPs) at segregating sites, in most cases assumed to be biallelic. They are often assessed in individuals sampled from study populations using targeted sequencing, genotyping arrays, or whole-genome sequencing, and they can range in number from a few to millions, depending on the organism and the technology used. Because alleles on the same chromosome are inherited together, it is common to refer to a specific combination of alleles at multiple SNPs that reside on the same chromosome as a haplotype.

Two powerful population genetic modeling frameworks are commonly used to model the genetic composition of a population, or multiple populations jointly. First, forward-in-time population genetic models, such as the Wright–Fisher model or diffusion (27, 71), describe the dynamics of population allele frequencies. These models account for fluctuations in population allele frequencies due to randomness in reproductive success, termed genetic drift. They can be extended to allow for the exchange of migrants in structured populations, model the dynamics of full genome sequences undergoing recombination, and readily include arbitrary mutation processes. They can also accommodate general fitness models to capture changes in genetic composition due to selection. Second, in backward-in-time models, the genetic composition of a sample of individuals or chromosomes from a population can be described using coalescent processes (52, 72, 145) by following the ancestral lineages of the sampled chromosomes into the past. These lineages coalesce when common genetic ancestors are reached, resulting in a genealogical tree that captures the ancestry of the sampled chromosomes. Again, the basic coalescent model can be extended to include the dynamics of full genomes undergoing recombination, general mutation processes, isolation or exchange of migrants in structured populations, and the effects of selection.

Inquiries into the processes that shape genomic variation have benefited tremendously from the development of tools that simulate either forward-in-time population models or coalescent processes. These tools are not the main subject of this review, but because they are used to gain insight into population genetic processes, to validate the methods that we discuss below, and to simulate data for simulation-based inference approaches, we briefly describe two prominent methods. One notable tool is the forward simulator SLiM, version 4 (SLiM 4) (48). Due to its flexibility, this tools excels at modeling nonneutral evolution in a variety of complex adaptation scenarios while allowing for detailed modeling of other population genetic processes. Another prominent simulation tool is the coalescent simulator msprime (3), which substantially extends the widely used simulation tool ms (58). msprime enables efficient simulation of genome-wide genetic variation for large samples under a variety of demographic scenarios and under a range of different models of evolutionary processes.

Another area of recent interest is the development of methods for the inference of ancestral recombination graphs (ARGs). In the coalescent framework, sampled chromosomes are related by a genealogical tree at each genomic position or locus, and the genealogical trees at different loci along the genome are correlated. These methods aim to infer these genome-wide multilocus genealogies (i.e., ARGs) directly from population genomic data. Because, in principle, they capture all ancestral events that affect the observed genetic variation, they are a very effective way to study the underlying population genetic processes. We highlight this type of inference here because some methods presented below require that ARGs be inferred as the first step. Methods to infer ARGs with high accuracy [e.g., ARGweaver (115)] are often applicable only to small samples, whereas other approaches [e.g., Relate (133) or tsInfer (67) combined with tsDate (149)] can be applied to thousands of samples.

The remainder of this review is organized as follows. In Section 2, we describe how different demographic processes shape the genomic variation in a population. We elaborate on how population genetic models can be used to describe various aspects of these underlying demographic processes and review methods that extract the corresponding signals from sampled data. Along similar lines, in Section 3, we present population genetic modeling approaches that characterize the dynamics of nonneutral genetic variants subject to selection, and we discuss methods that have been devised to detect these variants. We focus on selective sweeps of beneficial genetic variants and balancing selection. In Section 4.1, we briefly discuss situations in which the respective approaches can be used for inference and situations that warrant caution. In particular, demographic inference performs well when using neutral genetic variation, and outliers against a neutral background can be detected as candidates for adaptive genetic variation. However, results can be confounded when nonneutral processes affect genetic variation genome-wide, for example, in the form of background selection. In Section 4.2, we summarize recent approaches that aim to jointly characterize demographic models and genome-wide selection. Lastly, while not the primary focus of this review, in Section 4.3 we briefly describe how time-series genetic data, like ancient DNA data sets (100) or data from evolve-and-resequence experiments (122) in the laboratory, can improve inference. The scripts used to perform the simulations and create the figures for this review are available elsewhere (see https://github.com/steinrue/review_demo_sel_figs).

## 2. GENOMIC SCANS FOR DEMOGRAPHY

**Figure 1:**
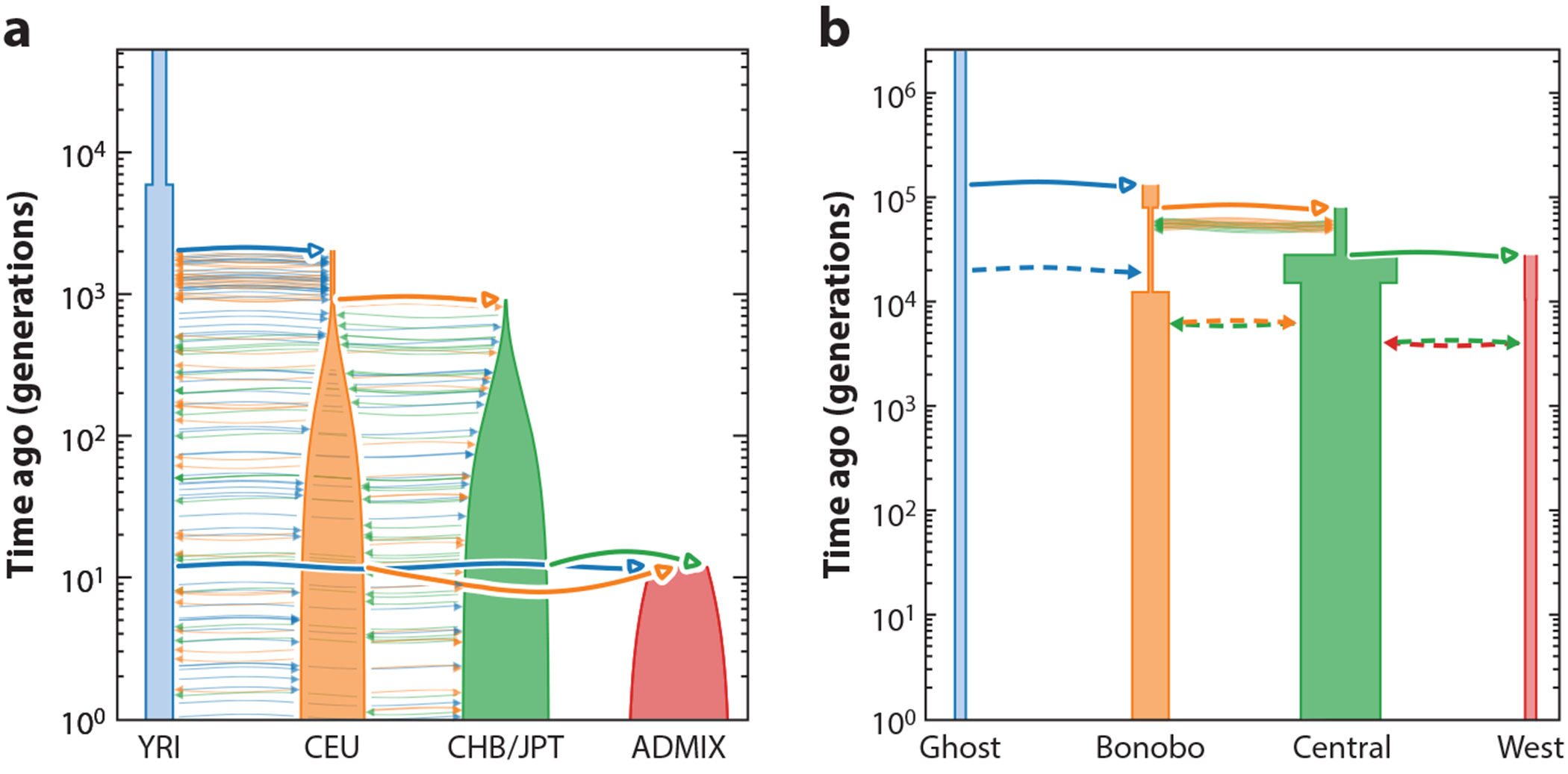
Examples of two complex demographic histories relating extant populations of varying sizes through migration and admixture. (*a*) A model of continental human populations [Yoruba in Ibadan, Nigeria (YRI); residents of Utah, USA, with European ancestry (CEU); Chinese in Beijing, China (CHB); and Japanese in Tokyo, Japan ( JPT)] and their admixture (ADMIX), presented by Browning et al. (11), who extended the model used by Gravel et al. (44). (*b*) A model of the demographic history of bonobos and Central/West African chimpanzees, presented by Kuhlwilm et al. (74). Both models are included in the stdpopsim catalog (77), which contains models for different species estimated in the literature for convenient simulation. The plots were created with the demes library (42), a standardized framework for estimating and sharing demographic models.

Throughout, we use the term demographic model or history relating to extant populations to refer to past effective population sizes (*N*_e_), divergence times of ancestral populations, historical migration rates between populations, and admixture or introgression events from source populations into target populations. Figure 1 shows two examples of such models from the literature. Methods to unravel this demographic history either directly infer specific parameters of the underlying demographic model or use nonparametric approaches to characterize aspects of this history. These approaches extract different signals from various types of data; thus, certain methods have more power in certain time frames and can resolve different aspects of the underlying demographic history.

### 2.1. Correlation of Allele Frequencies Across Populations

Nonparametric dimension-reduction techniques can visualize major axes of variation in genomic data and, thus, are widely employed to uncover the relationships between sampled individuals and their respective populations. In this context, principal component analysis (PCA) has become a standard approach. Genetic variation often results from isolation by distance; that is, individuals close to one another in geographic space are more likely to share recent ancestry. The major axes of genetic variation therefore tend to be aligned with geographic space (99), so the principal components can be used to visualize geographic structure in a population genomic data set. However, PCA is not free from artifacts—for example, there may be uneven sample size (91) and out-of-sample bias (80) that have to be accounted for when interpreting the results.

Other nonlinear dimension-reduction techniques that have been applied to genomic data include t-SNE (*t*-distributed stochastic neighbor embedding) (83) and UMAP (uniform manifold approximation and projection) (23), which place greater emphasis on local geographic structure. Model-based approaches, such as EEMS (estimating effective migration surfaces) (109) and fEEMS (fast estimation of effective migration surfaces) (90), fit fine-scale migration rates to the allele-frequency data, thereby allowing one to translate patterns in the data into conclusions about migration barriers in geographic space.

F-statistics, pioneered by Sewall Wright (150), are another widely applied class of methods used to reveal relationships between populations. They include the fixation index (F_ST_), which quantifies population differentiation, as well as F_3_- and F_4_-statistics (104), recently reviewed elsewhere (107), which quantify the phylogenetic relationships between populations. F_3_ and F_4_ in particular are often used to provide more rigorous statistical support for relationships hypothesized from exploratory approaches like PCA. In addition, the qpWave method (47, 87, 104) uses F-statistics to determine which populations form clades, and the qpGraph method (87, 104) uses them to estimate phylogenetic relationships between populations, including admixture events. These methods compute the F-statistics expected under a given demographic model and fit the model to the statistics observed in the data. The TreeMix method (110) follows a similar approach but differs in how it explores the space of possible phylogenetic relationships.

Another widely used class of approaches aims to directly characterize admixture events by identifying the source populations and mixture proportions that these sources contribute to admixed populations. Several of these approaches can be interpreted as versions of latent factor models (26) and, thus, have theoretical connections to PCA. Prominent examples are ADMIXTURE (2), which uses unphased genotype data, and fineSTRUCTURE combined with ChromoPainter (78), which uses phased haplotype data, to estimate the admixture components of focal population groups or individuals. qpAdm (49, 87) similarly estimates admixture proportions using F-statistics computed from genotype data.

### 2.2. Demographic Inference Using the Site-Frequency Spectrum

**Figure 2:**
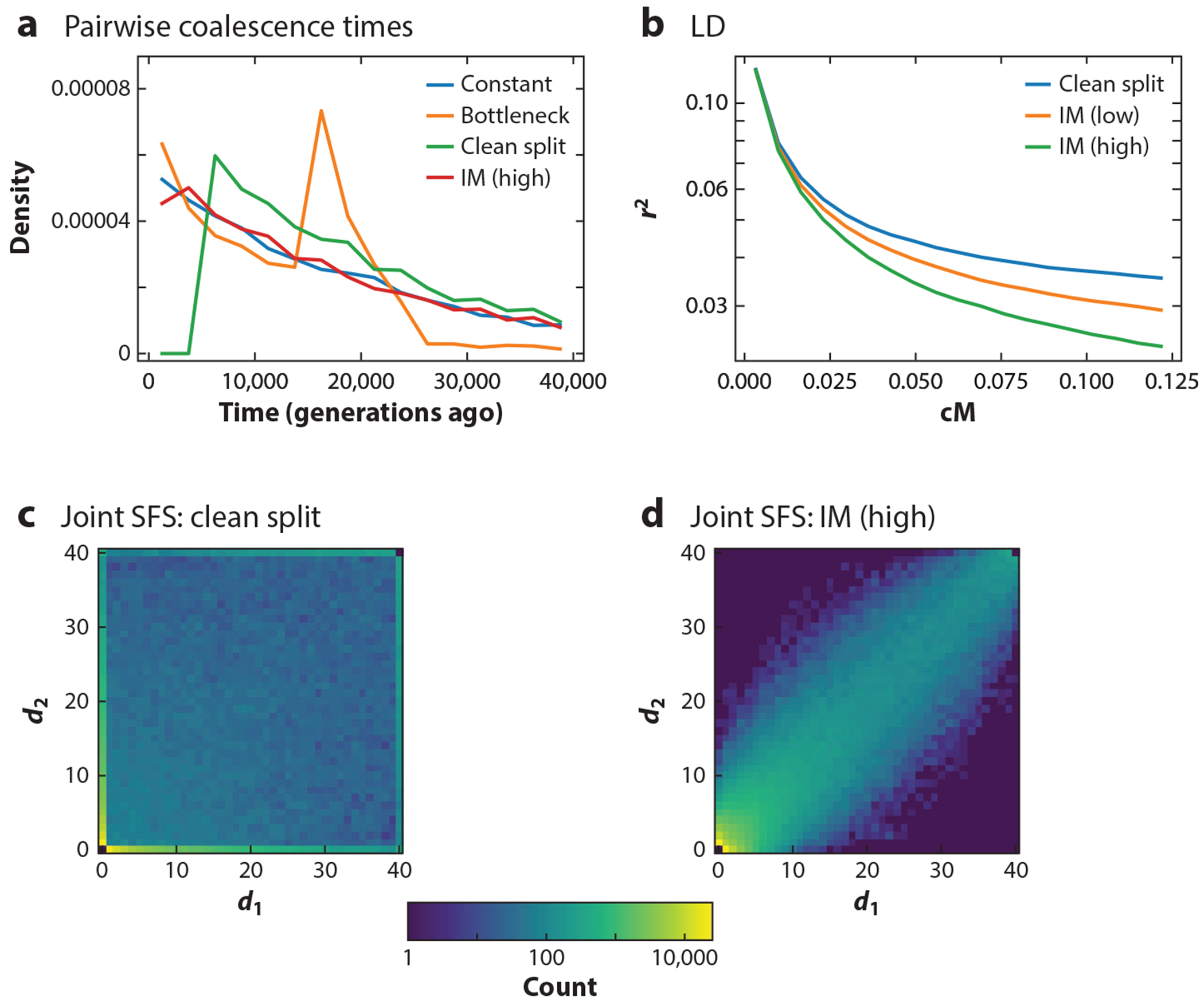
Signatures characteristic of demographic events in genomic data sets, simulated with msprime (3). (*a*) Density of pairwise coalescence times within and across extant populations. The density is higher during the bottleneck, and no coalescence occurs more recently than divergence in the clean split model. (*b*) LD among pooled samples extends further with increased isolation after divergence. Derived allele counts (*d_i_*) in extant populations are (*c*) largely uncorrelated in the joint SFS under the clean split model but (*d*) highly correlated when gene flow is strong. Constant refers to one population with *N*_e_ = 10,000. Bottleneck refers to *N*_e_ reduced to 2,500 between 15,000 and 25,000 generations before present. Clean split encompasses two extant populations of size *N*_e_ = 5,000 that diverged from an ancestral population of size *N*_e_ = 10,000 at generation 5,000 before present, with no gene flow between extant populations after divergence. IM is the same demographic model with gene flow after divergence: *m* = 5 × 10^−5^ per individual per generation (low) or *m* = 10^−3^ (high). Abbreviations: IM, isolation with migration; LD, linkage disequilibrium; *N*_e_, effective population size; SFS, site-frequency spectrum.

The site-frequency spectrum (SFS), or the joint SFS in the case of multiple populations, is a widely used summary of genomic data. It reflects the distribution of allele frequencies within and the sharing of alleles between populations, which provide information about the underlying effective population sizes and the history of gene flow, respectively (Figure 2c,d). Thus, many methods have been developed to estimate demographic models using the SFS. Most approaches compute the theoretical expectation of the SFS under a given demographic model by using the Poisson random field model (120) in either a diffusion-based or a coalescence-based framework. These expectations are then fitted to the observed data to infer the demographic history. The Stairway Plot 2 (84) and fastNeutrino (5) methods are designed to estimate the population size history of a single panmictic population from the SFS. These single-population methods can often be applied to samples of size 1,000 or more, allowing them to focus on events in the very recent past.

The joint SFS can be used to estimate demographic models that relate multiple extant populations. To obtain the expected joint SFS, ∂a∂i (46), moments (63), and momi2 (64) solve the allele-frequency dynamics for a specific demographic model by using different numerical approaches, whereas fastSimcoal2 (28) uses coalescent simulations to approximate the expected joint SFS. Again, the resulting expectations are then fitted to the SFS observed from the data to estimate parameters of the underlying demographic model. Note that most SFS-based methods implicitly assume that all of the segregating sites are unlinked and evolve independently. G-PhoCS (45), IMa2p (124), and MIGRATE (4), however, model the data in several small, unlinked genomic regions, sufficiently far apart, as independent, and they assume complete linkage within each region.

### 2.3. Inferring Demographic Models Using Linkage Patterns

Close relatives share long genomic segments that are identical by descent (IBD), and ancestral recombination events break them into shorter segments if the relation is more distant. Consequently, linkage disequilibrium (LD) extends further along the genome when individuals share more recent ancestry, and the resulting decay pattern carries substantial information about the demographic history (e.g., Figure 2b). Thus, many methods have been developed to infer demographic history from cumulative genome-wide LD statistics, that is, LD statistics averaged over many pairs of loci. For accurate inference, LD-based methods require a sufficiently accurate genetic map of recombination rates.

Expectations of observed LD statistics can be obtained by computing the decay of LD under a given demographic model relating multiple populations. These expectations can then be fitted to the statistics observed in the data for inference. Ragsdale & Gutenkunst (113) and the moments.LD method (112) compute these expectations by solving equations describing the evolution of genetic variation at two linked loci under a given demographic model numerically. Modeling the decay of LD in scenarios where a given population is admixed from two source populations allows ALDER (85) to focus on estimating the admixture proportions and ROLLOFF (94) to estimate the time of admixture.

The patterns of long IBD segments in a population genomic data set can also be used directly to provide another lens to unravel the demographic history. Most IBD-based methods first identify these segments, often approximating them using identity by state (IBS), since true IBD cannot be observed in the data. They then compare the observed distribution with distributions expected under a certain demographic model. Because long shared segments indicate recent common ancestry, these methods provide information about recent demographic events, especially in the last 100 generations before the present, but tend to require a large sample size in order to observe sufficiently many shared segments.

The IBDNe method (10) infers the history of effective population sizes in a single population on the basis of IBD sharing in the sample. DoRIS (102) focuses on estimating divergence times between two extant populations, and Tracts (43) characterizes the admixture history of a population admixed from two sources. GLOBETROTTER (54), combined with ChromoPainter (78), can characterize admixture with multiple admixture events from different sources. Moreover, Ralph & Coop (114) used sharing of long genomic segments to elucidate recent gene flow across Europe, and modeling migration rate variation in space rather than gene flow between discrete populations allowed MAPS (1) to estimate local migration rates across geographical space from patterns of shared genomic segments.

### 2.4. Coalescence-Based Inference Approaches

From a genealogical perspective, the signals used for demographic inference are the rates at which ancestral lineages within and between populations coalesced at certain times in the past. Within a given population, these rates are inversely proportional to its effective size, *N*_e_, and the rates between populations indicate gene flow (e.g., Figure 2a). A very powerful class of methods based on coalescent hidden Markov models (CHMMs), recently reviewed elsewhere (134), infer demographic histories from whole-genome sequencing data. These methods capitalize on the correlation between genealogical trees relating the sampled chromosomes at different loci. This correlation structure is well approximated by a Markov chain. Thus, the demographic inference can be cast as a CHMM, where the unobserved genealogies at each locus are the hidden states and the observed genetic variations are the emissions. The distribution of the hidden states depends on the parameters of the underlying demographic model, which can thus be inferred using expectation–maximization approaches.

Available methods differ by (*a*) the sample size to which they can be applied and (*b*) the representation of the genealogical trees in the inference framework. To infer the effective population size history of a single panmictic population, the widely applied PSMC method (82) utilizes genomic data from one unphased individual, diCal (126) uses the coalescence time of a distinguished haplotype in a composite leave-one-out approach, and SMC++ (140) uses the coalescence time of two distinguished haplotypes from the sample.

If the data originate from multiple populations, methods take either a parametric approach that estimates the parameters of an underlying demographic model or a nonparametric approach that characterizes the varying intensity of gene flow between past populations. The diCal2 (135), IMCoalHMM (16), and Jocx (17) methods implement the parametric approach. diCal2 uses a leave-one-out representation of the local genealogies that is similar to that of diCal (126), and IMCoalHMM and Jocx integrate over the full genealogies of a small sample in scenarios involving two or three populations. Parameter-free approaches include MSMC (121), which represents genealogies using the first coalescence time in the sample, and MSMC2 (146), which uses pairwise coalescence times between all samples as representation.

Moreover, as described in Section 1, several methods have recently been developed to infer multilocus genealogies (ARGs) from genomic data. Given the underlying genealogy, the coalescence events and, therefore, the coalescence rates can be directly measured, with some uncertainty, and used to gauge the underlying demographic events. Several recent studies have used this approach to investigate the demographic history of modern humans (133, 149).

### 2.5. Machine Learning Approaches Based on Simulated Data

The increase in computing power has led to the emergence of simulation-based approaches to demographic inference. Rather than analytically or numerically computing the expectations of summary statistics for genomic data, one can obtain these expectations from simulations of the underlying population genetic processes. In order to infer the parameters of a demographic model, a large number of replicates are simulated under different parameter values and supervised machine learning models are trained on statistics of the simulated data to identify the respective demographic parameters. These trained models are then applied to the real data to obtain estimates of the parameters. Methods differ in the type of summary statistics they use and in the specific type of machine learning approach.

A popular framework implementing this approach is approximate Bayesian computation (ABC). ABC has been applied to statistics characterizing nucleotide diversity *π* to infer parameters of demographic models that relate multiple populations (31), diversity- and haplotype-based statistics to characterize gene flow between two populations (130), and population size history of a single population from the full SFS and LD statistics using the PopSizeABC method (8). Moreover, generative adversarial networks (e.g., pg-gan; 147) and convolutional neural networks (35) have been employed to infer gene flow between two populations through the use of whole-genome data.

## 3. GENOMIC SCANS FOR NATURAL SELECTION

Natural selection can change the population frequency of genetic variants that alter the fitness of an organism over time. Positive selection tends to increase the frequency of beneficial alleles, whereas negative selection tends to decrease the frequencies of unfavorable alleles. Selection can also act to maintain polymorphism at a focal locus, referred to as balancing selection. In this section, we focus on methods that use population genomic data to detect genetic variants under positive selection or balancing selection. Negative or purifying selection is often assessed with sequence conservation between species (151), but some recent approaches incorporate genomic variation within species (57). In Section 4, we discuss how purifying selection and background selection, caused by purifying selection on linked variants, affect genome-wide genetic variation.

### 3.1. Positive Selection

In the following subsections, we discuss different types of dynamics for beneficial alleles and methods to detect the resulting signatures, as well as approaches to detect selection on the basis of differentiation of allele frequencies between populations.

#### 3.1.1. Complete selective sweep of a de novo variant.

**Figure 3:**
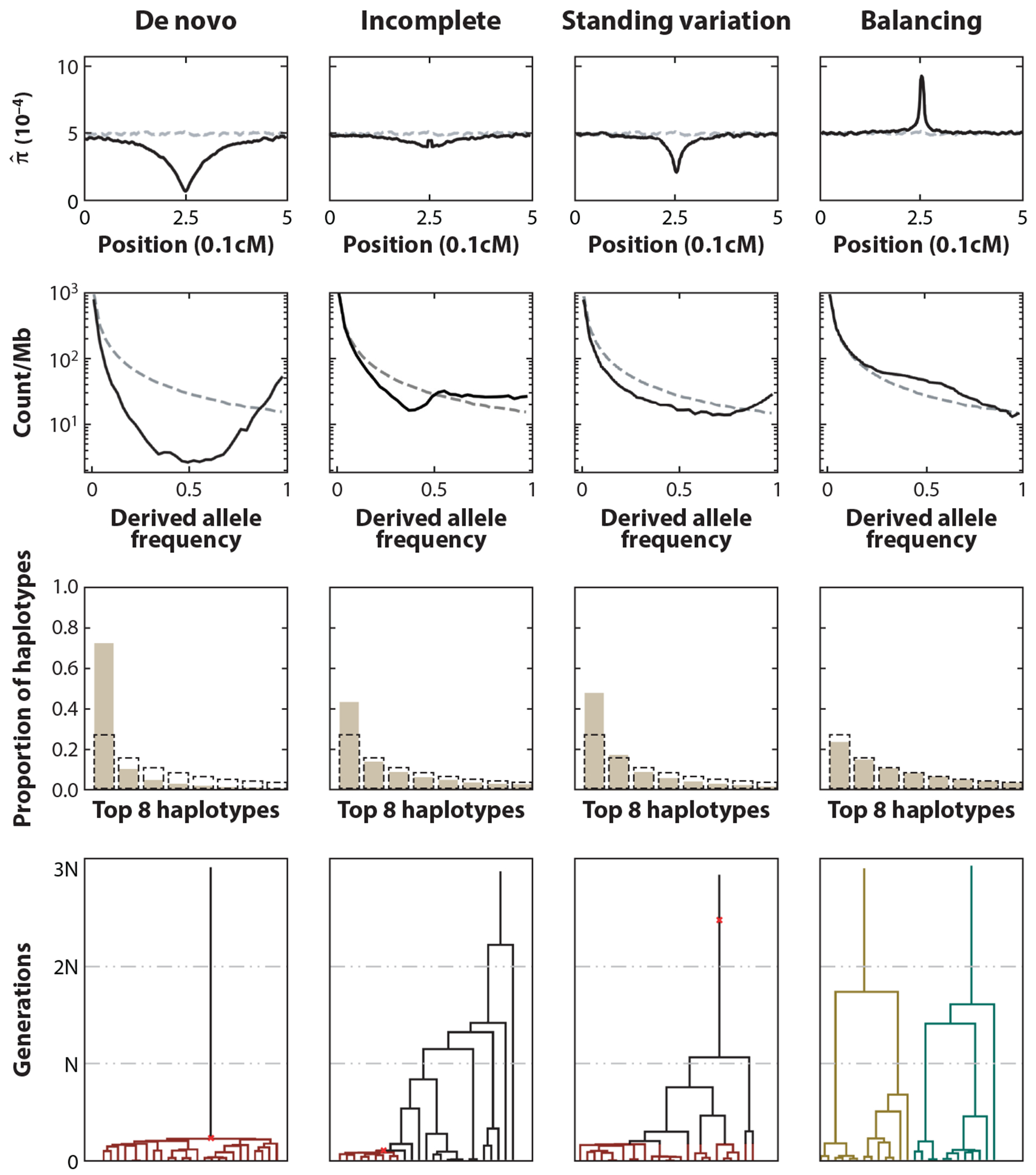
Genomic footprints of four modes of selection, simulated with SLiM 4 (48). Each column corresponds to a particular type of selection. Each row represents a particular feature of genetic variation and the corresponding patterns as observed in simulated data. (*Top to bottom*) Mean nucleotide diversity *π* (per 10 kbp window), SFS of the 2 kbp window centered on the selected locus, relative abundances of the eight most common haplotypes in a 10 kbp window (HFS), and the local genealogical trees of 25 randomly selected lineages at the selected locus. Gray dashed lines indicate the average level in neutral replicates. Abbreviations: HFS, haplotype-frequency spectra; SFS, site-frequency spectra.

When a new, strongly beneficial mutation is introduced into a population and is not immediately lost due to genetic drift, its frequency increases rapidly until fixation with high likelihood. Closely linked neutral alleles on the same haplotype as the beneficial allele will also increase in frequency, a phenomenon termed genetic hitchhiking (131), whereas the frequency of alleles on other haplotypes will decrease. This effect is weaker for loosely linked alleles at greater genomic distances, since their dynamics will be decoupled by recombination over time and haplotypes will be shortened. Thus, in proximity to the locus with the beneficial allele, genetic diversity is reduced and one haplotypic background dominates. These effects lead to an excess of high-frequency derived alleles in the local SFS of the genomic region (Figure 3). From a genealogical perspective, ancestral lineages linked to the adaptive allele coalesce quickly when traced back in time, resulting in low genetic diversity. In contrast, they follow the neutral coalescent dynamics if recombination decouples them from the adaptive allele (66). The quick fixation of a beneficial de novo variant is also referred to as a hard sweep.

Since one haplotypic background dominates after a completed sweep, LD extends further than under neutrality (Figure 3). The extended haplotype homozygosity (EHH) (117) quantifies this signal to identify sweeps using the average pairwise homozygosity along the genome in samples carrying the focal allele. The integrated haplotype score (iHS) (143) extends this approach by suitably integrating the EHH and contrasting the integrals for the derived and the ancestral allele. Moreover, when a sweep is completed, the excess LD does not extend across the locus with the beneficial allele. The statistic *ω* (70) thus locates completed selective sweeps by characterizing excess LD on either side of a potential locus under selection. Capitalizing on the skewed distribution of allele frequencies, SweepFinder2 (21) computes the composite likelihood of the local SFS under an empirical neutral distribution and contrasts it to the likelihood of the local SFS being distorted due to a recent sweep. The resulting composite likelihood ratio (CLR) statistic can then be used to assess evidence for a recent sweep. In a similar framework, SweeD (106) uses analytic results to compute the neutral SFS.

#### 3.1.2. Sweeps from standing variation and incomplete sweeps.

In the scenario described in the preceding subsection, a de novo variant fixes rapidly. Deviations from this scenario lead to more subtle genomic footprints and are often referred to as soft sweeps (55). First, in an incomplete or partial sweep, the new variant loses its selective advantage before fixation occurs, and the haplotype carrying the beneficial mutation does not fix. Thus, the diversity is not reduced as substantially as during a complete sweep (Figure 3). Second, a sweep from standing genetic variation occurs if a genetic variant is segregating in the population before becoming beneficial and sweeping to fixation, for example, when the environment changes. Alternatively, two or more beneficial mutations can occur in close proximity at around the same time. Since beneficial alleles then reside on multiple haplotypic backgrounds, several haplotypes can be at appreciable frequencies at the end of the sweep.

To detect incomplete sweeps, Vy & Kim (144) extended the SweepFinder2 model (21) to account for the distortion of the SFS in this scenario. Furthermore, the statistic nS_L_ (33) measures homozygosity similarly to iHS (143), and nS_L_ and iHS retain substantial power to detect incomplete sweeps and sweeps from standing variation (33). Additionally, several approaches have been developed on the basis of the haplotype-frequency spectrum (HFS) in a genomic window. The haplotype homozygosity in a genomic window is given as the sum of squared haplotype abundances. Homozygosity computed when combining the two most abundant haplotypes (H12) is elevated after a completed sweep. Moreover, the ratio of the homozygosity excluding and including the most abundant haplotype (H2/H1) quantifies the number of overabundant haplotypic backgrounds and, thus, can be used to distinguish between a sweep from de novo mutation and a sweep from standing variation (40) (Figure 3). Lastly, the LASSI method (50) introduces a CLR test similar to SweepFinder2 (21). Here, the likelihood of the HFS in a small genomic window under the empirical neutral distribution is compared with the likelihood of the HFS distorted by a sweep, where the window is dominated by a small number of haplotypes. The number of haplotypes is estimated to provide flexibility and distinguish different sweep scenarios.

#### 3.1.3. Detecting positive selection using excess differentiation between populations.

When data from multiple populations is available, excess frequency differentiation at a particular locus can indicate selection, possibly due to local adaptation. This signal can be especially effective for weak or old selective sweeps, where the genomic footprints of hitchhiking have been eroded by mutation and recombination.

As discussed in Section 2.1, the fixation index F_ST_ (150) is frequently used to measure genetic differentiation between populations. Genome-wide F_ST_ reflects the extent of genetic drift or the length of the phylogenetic branches between extant populations. Interpreting pairwise −log(1 − F_ST_) as phylogenetic branch length more directly (116), the PBS (population branch statistic) (152) identifies specific loci with exceptionally long branches in a phylogeny relating three populations, suggesting positive selection in the respective population.

By directly using F_ST_ between multiple populations, methods such as the FLK test (9), the LRT statistic (6), BayeScan (36), and Bayenv (20) identify statistically significant outlier loci from the genome-wide null distribution as candidates for selection. Extending this approach to better account for correlated frequency changes at linked loci due to genetic hitchhiking, Flink (38) models correlations of locus-specific differentiation in close proximity. Moreover, XP-CLR (15) and 3P-CLR (111) define a composite likelihood test that explicitly models the population allele-frequency differentiation at neutral loci linked to a selective sweep, in addition to the differentiation at the selected locus.

Assessing haplotypic backgrounds more explicitly, XP-EHH (118) contrasts the EHH (117) between populations, where excess levels indicate a selective sweep in the respective population. Similarly, *χ*_MD_ (76) contrasts haplotype sharing within different populations in genomic regions, and the SS-H12 method (51) examines the two most abundant haplotypes at a locus in two populations to distinguish two separate sweeps in the extant populations from a shared ancestral sweep.

Many approaches to detecting selection using population differentiation require discrete population labels for the sampled individuals, which are not always available. However, as discussed in Section 2.1, dimension-reduction techniques can be used to capture population structure in unlabeled data. Thus, PCAdapt (86) identifies the principal components in the data by using PCA and estimates the loadings of each locus on these components. High values for a particular locus indicate excess differentiation at this locus along major axes of variation, a proxy for population label, which indicates selection. Similarly, TESS3 (12) uses latent factor models to assess excess differentiation at particular loci.

### 3.2. Balancing Selection

Balancing selection maintains polymorphism at target loci through various mechanisms (13). Over longer timescales, balancing selection thus maintains multiple short haplotypes, causing an enrichment of alleles at intermediate frequencies. From a genealogical perspective (65), ancestral lineages at neutral loci linked to different selected alleles can coalesce only after recombination places them into the same genetic background, resulting in deep coalescent trees (Figure 3). Thus, the characteristic signatures of long-term balancing selection are narrow genomic regions of high genetic diversity around the selected locus, an increased number of segregating sites, and a local SFS that is enriched for alleles at intermediate frequencies. The size of the affected region will be substantially smaller than for a selective sweep, since recombination acts on longer timescales.

The BALLET method (22) computes CLR statistics that contrast the likelihoods in genomic windows under a model of balancing selection and empirical neutrality to identify regions that indicate balancing selection. This statistic performs well in different demographic scenarios (22). Moreover, several statistics have been developed to quantify the excess of alleles at intermediate frequencies around a locus under balancing selection. Noncentral deviation (7) tests for deviation from an intermediate frequency, and lower values than expected under neutrality provide evidence of balancing selection. Similarly, BetaScan2 (128) weighs SNPs in the local SFS according to their frequency to derive a test statistic indicative of balancing selection.

If the allele under balancing selection predates speciation, it can lead to trans-species polymorphisms (TSPs). Gao et al. (39) extend the coalescent framework for balancing selection (65) to investigate expected patterns of LD and allele sharing around TSPs under balancing selection in scenarios with two closely related species. Similarly, MULLET (18) extends the CLR test of BALLET (22) to multiple species and includes nucleotide substitutions between the species in the likelihood model.

Most methods require specification of a genomic window size to compute statistics or assume a single site to be under balancing selection. If selection acts at multiple sites epistatically to maintain polymorphism, then the size of the affected region can be larger (97). To account for the variation in footprint size, BalLeRMix (19) implements a CLR test based on a mixture model that combines the SFS expected under strong balancing selection with the empirical SFS at a linked neutral locus. The mixture weights, corresponding to recombination distance and thus window size, are estimated to provide flexibility.

### 3.3. Genealogical Approaches to Detect Selection

Many methods covered in the preceding sections use genetic variation to implicitly assess the distortion of local genealogies due to selection. Several approaches more explicitly infer features of the underlying genealogy, or the multilocus genealogy (ARG) directly, to characterize positive or balancing selection. A genealogical feature that indicates selection is the length of the branches in the tree. Short external branches that coalesce quickly in the genomic background carrying the beneficial allele indicate recent positive selection, whereas long branches are indicative of balancing selection (Figure 3).

The singleton density statistic (SDS) (34) infers the length of external branches in genomic regions by considering the genomic distance of a focal variant to singleton mutations. High values indicate short external branches on a focal allelic background, which in turn indicate recent positive selection. Other methods that explicitly infer the local coalescence times between pairs of sampled chromosomes include tsel (59), using the software PSMC (82), and ASMC (103), using a similar framework as SMC++ (140). Statistical assessment of deviations from the genome-wide distribution of pairwise coalescence times is then used to detect regions of positive selection (short branches) or balancing selection (long branches).

While most ancestral lineages carrying the beneficial allele coalesce quickly, ancestral recombination events decouple some lineages, and these coalesce with the rest of the genealogical tree further in the past. Lineages at loci with increasing genomic distance are more readily decoupled. Therefore, the resulting imbalances in the genealogical tree near a selected locus are another signature of positive selection. This topological signature does not depend on the length of the branches, and thus is more resistant to population size changes, but it may be affected by population structure. MFDM (81) uses the derived allele frequencies in a genomic region to bound the size of the subtrees descending from the most recent common ancestor of the sample and assesses evidence for positive selection by comparing these sizes with expectations under neutrality. bsfs or btree (24) quantifies imbalance at the internal nodes of a genealogical tree from the local SFS or an inferred ARG, respectively. A large imbalance indicates positive selection, whereas trees that are more balanced than neutral trees provide evidence for balancing selection.

In addition to presenting the method Relate to infer ARGs, Speidel et al. (133) introduce a statistic to assess tree imbalance on the basis of the inferred local genealogy. Deviation from neutral expectation provides evidence of positive selection, and these authors show improved performance of their statistic over SDS (34). CLUES (136) uses inferred multilocus genealogies (115) to estimate the selection coefficient and the allele-frequency trajectory of a beneficial allele at a given locus, using the coalescence rates of the ancestral lineages on the beneficial background. This method integrates over uncertainty in the estimated genealogy by using importance sampling.

### 3.4. Detecting Selection Using Machine Learning

As is the case for demographic inference, recent developments in data simulation and machine learning have inspired several methods to detect and quantify selection. In these approaches, genomic data are simulated under neutral evolution and different modes of selection, often under a target demographic model. Supervised machine learning approaches, such as artificial neural networks, are trained to classify the simulated data, and are subsequently applied to real data, to identify genomic regions as well as the mode and strength of selection.

Several approaches represent genomic data by using summary statistics that are sensitive to different aspects of nonneutral evolution. These summary statistics include statistics characterizing nucleotide diversity *π*, SFS-based neutrality tests [e.g., Tajima’s *D* (139), Fu & Li’s *D* (37), or Fay & Wu’s *H* (32)], LD statistics (see Section 2.3), and haplotype statistics sensitive to signals of selection (see Sections 3.1.1 and 3.1.2). Methods differ in the exact set of statistics used. In most approaches, these statistics are computed in several genomic windows, around 10–20 kbp in size, at and in proximity to the putatively selected locus. These statistics are then combined as feature vectors that are used to train different machine learning architectures, which can be applied in different scenarios. For example, Peter et al. (108) distinguish between sweeps from de novo and standing variation, Pavlidis et al. (105) identify complete sweeps from de novo variants, Sugden et al. (137) present the method SWIF(r) to detect complete and incomplete sweeps, and Sheehan & Song (127) present the method evoNet to identify balancing selection in addition to sweeps. SURFDAWave (95) more explicitly captures correlation among summary statistics across genomic windows by using the coefficients of wavelet representations as features to distinguish sweeps from de novo or standing variation.

Alternatively, raw genomic data can be interpreted as an image, where the rows are the sampled haplotypes and the columns are the SNPs. Such genomic images retain correlation structure across loci, and use of the raw data in this form allows machine learning approaches to extract important features of the data, rather than relying on prespecified summary statistics. ImaGene (141) employs this approach to detect selective sweeps and estimate their strength, and diploS/HIC (68) uses it to distinguish sweeps from de novo or standing variation using unphased data. Lastly, SIA (53) represents genomic data using explicit genealogies inferred by Relate (133) to identify and infer the selection strength and frequency trajectory of beneficial alleles using recurrent neural networks.

## 4. DISCUSSION

### 4.1. Methods for Demographic Inference or Identification of Selected Variants

Most of the methods for inferring demographic models presented in Section 2 work under the assumption that the genome-wide genetic variation evolves neutrally and is affected only by the demographic history. Neutral simulations under various demographic models, performed in the original studies or in later studies evaluating these methods, have demonstrated that the methods perform accurately in their respective scenarios. Thus, these methods are generally well suited for the characterization of demographic models when the assumption of neutrality is met. Consequently, they are often applied to putatively neutral genomic variation, such as nongenic regions or synonymous sites, or in a genome-wide fashion, in species where nonneutral evolution is generally believed not to substantially affect genome-wide variation. Establishing the validity of the latter assumption in different organisms is difficult, however, and general consensus is not always given.

The methods used to scan for nonneutral genetic variation at a specific locus, presented in Section 3, aim to identify patterns in the data that deviate from a theoretical or empirical genome-wide neutral distribution. In many cases, the studies presenting these methods include a discussion of their performance under different demographic scenarios, often in the form of simulations documenting the methods’ statistical properties for different models. Furthermore, some methods are designed specifically to be more resilient to demographic effects, for example, those based on the topology of genealogical trees. Other methods leverage genetic variation collected in different population groups to detect selection (see Section 3.1.3). Thus, researchers can use many approaches to detect genetic variation under selection at specific genomic loci, although simulation studies in specific scenarios should be performed.

### 4.2. Selection Affecting Genome-Wide Variation

**Figure 4:**
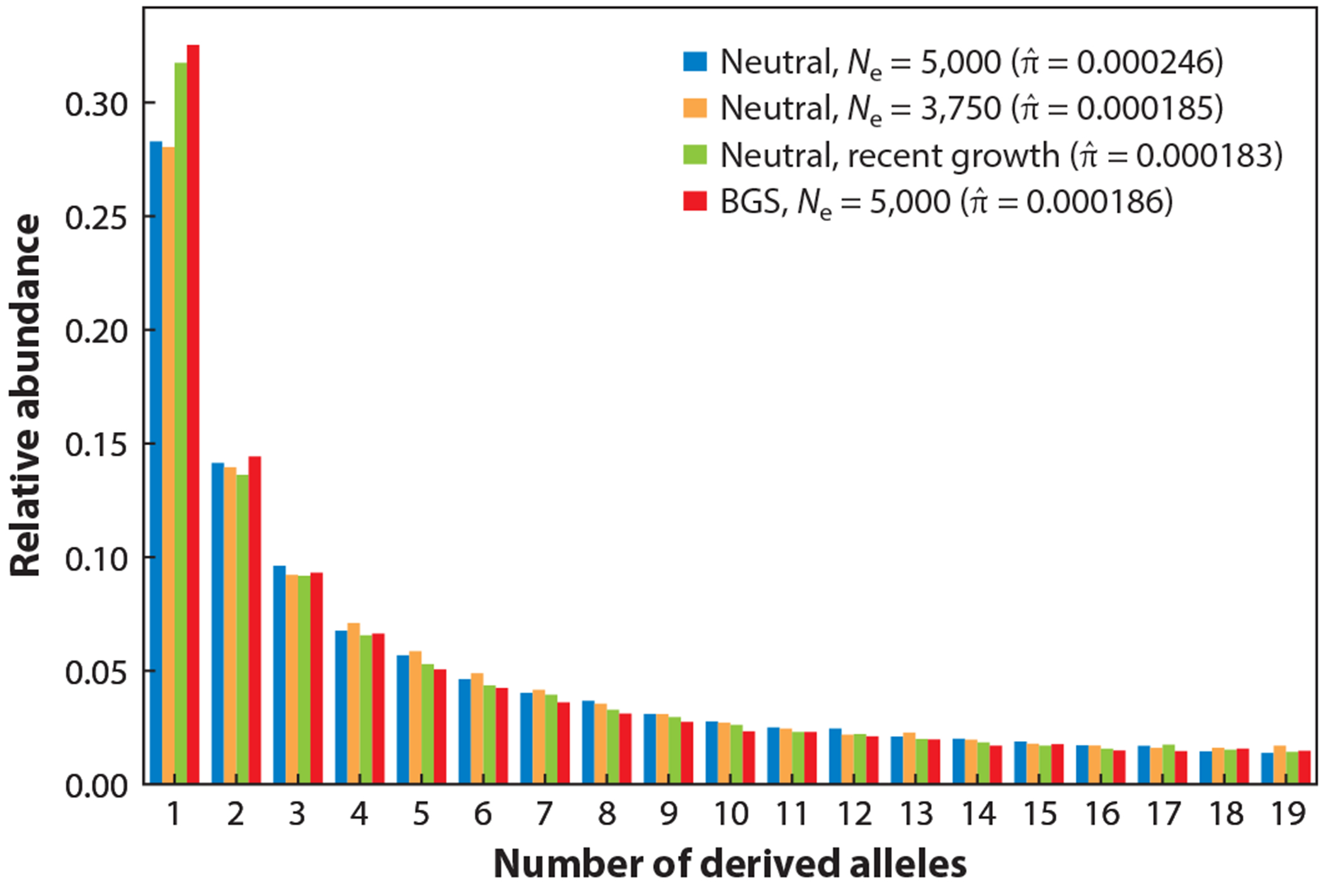
SFS simulated with SLiM 4 (48) under neutrality and a simplified model of background selection. The neutral model with *N*_e_ = 5,000 has the highest pairwise nucleotide diversity $\hat{\pi }$ . However, the neutral model with *N*_e_ = 3,750, the BGS model with *N*_e_ = 5,000, and a model of recent population growth yield approximately the same value of $\hat{\pi }$ . The BGS model produces more singletons in the SFS than a neutral model with constant *N*_e_; however, recent growth leads to a similar pattern. The BGS model does lead to a slight depletion among low- to medium-frequency variants, but it is challenging to choose a model on the basis of $\hat{\pi }$ or the SFS alone. Abbreviations: BGS, background selection; *N*_e_, effective population size; SFS, site-frequency spectra.

Challenges arise when considering forms of selection that affect genetic variation genome-wide, where the signals of selection cannot be considered outliers against a neutral genomic background. Especially notable here is direct purifying selection against deleterious mutations, which can be quantified by the distribution of fitness effects (DFE) (30). The DFE describes the sign and magnitude of the fitness effect of new nonneutral mutations. Many studies have aimed to estimate the DFE in different organisms, either experimentally or by use of genomic data from population samples (30). Furthermore, selection can also have indirect effects on putatively neutral genetic variation as a result of genetic hitchhiking. Purging of deleterious mutations from the population manifests as background selection (14, 92), generally reducing neutral genetic diversity (Figure 4). Diversity is also reduced by frequent, recurrent, selective sweeps of beneficial mutations (25, 56, 123). In many cases, selection also acts on complex phenotypes (119) that are affected by many genetic variants with small individual effects. Therefore, directional or stabilizing selection on these polygenic traits, or on correlated traits, also shapes the genome-wide patterns of genetic variation (29, 129, 153).

Thus, assuming neutrality when inferring a demographic model, even when using putatively neutral sites, can lead to biased inference (62). In contrast, when investigating how background selection or adaptation of complex traits shapes genetic variation, complex demographic models must be taken into account. Thus, ideally, the demographic model and the genome-wide distribution of direct and indirect selection would be estimated simultaneously (60).

Because population genetic models that account for these effects jointly are complex, it is difficult to develop methods that perform such joint inference efficiently and accurately (e.g., Figure 4), although some recent approaches have tackled this challenge. To estimate the DFE for direct fitness effects, the Fit∂a∂i method (69) infers population size history from the SFS of putatively neutral synonymous sites, then conditions on this history when estimating the DFE using nonsynonymous variation. Another approach (101) estimates the DFE and population size history on the basis of patterns of haplotype sharing around variants that segregate at low frequencies. Furthermore, in two studies attempting to explain the variation in genome-wide diversity levels in *Drosophila melanogaster* (25) and humans (96), the authors fitted a model that accounts for different fitness parameters for adaptive substitutions and background selection, using detailed genome annotations for nucleotide substitutions and conserved genomic regions, respectively. Similarly, Johri et al. (61) used diversity and linkage statistics computed in genomic windows for exonic data in an ABC framework to jointly estimate a demographic model and the fitness parameters for purifying and background selection in humans. These examples demonstrate that, in order to elucidate the evolutionary processes underlying genetic variation, large genomic data sets must be analyzed with carefully designed methods based on comprehensive models incorporating the joint effects of different population genetic processes.

### 4.3. New Perspectives from Time-Series Genetic Data

Throughout this article, we have presented approaches that aim to unravel demographic models and natural selection using contemporary population genomic data. These methods use patterns of genomic variation in contemporary populations to gauge how the genetic composition changed in the past and assess the forces underlying these changes. In recent years, researchers have collected large amounts of ancient DNA (i.e., genetic material extracted from deceased individuals) in humans as well as in other species (73, 100, 125). Similarly, researchers have assessed the genetic composition of organisms evolving in the laboratory over time, subject to controlled environmental conditions and selective pressure (41, 122). This type of time-series genetic data presents novel perspectives for population genetic inference: Instead of indirectly inferring changes in the genetic composition of past populations, these data sets allow direct observation of the changes and, thus, have the potential to elucidate adaptive and demographic forces more comprehensively. Such data sets have already refined our understanding of recent human demographic history (79) and allowed us to explore the emergence and frequency trajectories of adaptive genetic variation in greater detail (89).

However, these data sets do not come without challenges. In contemporary populations, high-quality sequencing data can often be obtained in humans and model organisms, but even in nonmodel organisms technological advances are continuously improving the quality of the data. However, when using ancient DNA, for example, the sampling scheme cannot be designed, and researchers are restricted by the availability of samples. The amount of genetic material to process is limited, fragmented, and damaged (100). Data quality is steadily improving as a result of technological and computational advances, but limitations remain. These limitations are especially relevant because many of the approaches described in this review benefit from the identification of characteristic patterns of LD in the data, which is challenging to assess in ancient DNA [although recent methodological improvements are promising (132)]. We note that some of the methods to characterize demographic models based on allele-frequency correlations presented in Section 2.1 are also frequently used to analyze ancient DNA.

In summary, many methods can accurately perform demographic inference or scan for specific genetic variants under selection in a variety of scenarios. However, unraveling the interplay among the evolutionary processes that shape genetic variation genome-wide will require the development of inference frameworks that can accurately characterize the demographic history and nonneutral processes jointly and make efficient use of modern data sets.

## Abbreviations

Single-nucleotide polymorphism (SNP): genomic position where different nucleotides are observed in a sample
Haplotype: specific combination of alleles at multiple SNPs that reside on the same chromosome
Ancestral recombination graph (ARG): graph structure comprising genealogies relating samples at multiple loci
Locus: refers to either a genomic position or a genomic region of interest
Selective sweep: an allele conferring a fitness advantage quickly increases in frequency until fixation
Demographic model: comprises past effective population sizes, divergence of ancestral populations, migration events, and population structure
Fixation index (F_ST_): measures differentiation of allele frequencies between populations
Site-frequency spectrum (SFS): histogram of the derived allele counts observed across sites
Joint SFS: tabulates the number of combinations of derived allele counts observed in multiple populations across sites
Poisson random field model: assumes that SNPs result from single mutations that evolve independently
Identity by descent (IBD): genomic segment inherited from a common ancestor, uninterrupted by recombination
Linkage disequilibrium (LD): nonrandom association between alleles at a pair of loci
Identity by state (IBS): genomic segment where two chromosomes carry the same alleles
Nucleotide diversity *π*: number of sites at which two haplotypes differ, averaged over all pairs
Genetic hitchhiking: frequency changes of a selected allele affect neutral alleles as a result of chromosomal linkage
Homozygosity: sum of squared frequencies of all alleles at a given locus (SNP or genomic region)
Haplotype-frequency spectrum (HFS): tabulates the abundances of unique haplotypes (allelic combinations) in a given genomic window
SFS-based neutrality tests: test the compatibility of the SFS with neutral evolution

## References

[1] Al-Asadi H, Petkova D, Stephens M, Novembre J. 2019. Estimating recent migration and population-size surfaces. PLOS Genet. 15(1):e1007908 30640906 10.1371/journal.pgen.1007908 PMC6347299

[2] Alexander DH, Novembre J, Lange K. 2009. Fast model-based estimation of ancestry in unrelated individuals. Genome Res. 19(9):1655–64 19648217 10.1101/gr.094052.109 PMC2752134

[3] Baumdicker F, Bisschop G, Goldstein D, Gower G, Ragsdale AP, 2022. Efficient ancestry and mutation simulation with msprime 1.0. Genetics 220(3):iyab229 34897427 10.1093/genetics/iyab229 PMC9176297

[4] Beerli P, Mashayekhi S, Sadeghi M, Khodaei M, Shaw K. 2019. Population genetic inference with MIGRATE. Curr. Protoc. Bioinform 68(1):e87 10.1002/cpbi.87 PMC9286049 31756024

[5] Bhaskar A, Wang YXR, Song YS. 2015. Efficient inference of population size histories and locus-specific mutation rates from large-sample genomic variation data. Genome Res. 25(2):268–79 25564017 10.1101/gr.178756.114 PMC4315300

[6] Bhatia G, Patterson N, Pasaniuc B, Zaitlen N, Genovese G, 2011. Genome-wide comparison of African-ancestry populations from care and other cohorts reveals signals of natural selection. Am. J. Hum. Genet 89(3):368–81 21907010 10.1016/j.ajhg.2011.07.025 PMC3169818

[7] Bitarello BD, De Filippo C, Teixeira JC, Schmidt JM, Kleinert P, 2018. Signatures of long-term balancing selection in human genomes. Genome Biol. Evol 10(3):939–55 29608730 10.1093/gbe/evy054 PMC5952967

[8] Boitard S, Rodríguez W, Jay F, Mona S, Austerlitz F. 2016. Inferring population size history from large samples of genome-wide molecular data—an approximate Bayesian computation approach. PLOS Genet. 12(3):e1005877 26943927 10.1371/journal.pgen.1005877 PMC4778914

[9] Bonhomme M, Chevalet C, Servin B, Boitard S, Abdallah J, 2010. Detecting selection in population trees: the Lewontin and Krakauer test extended. Genetics 186(1):241–62 20855576 10.1534/genetics.110.117275 PMC2940290

[10] Browning SR, Browning BL. 2015. Accurate non-parametric estimation of recent effective population size from segments of identity by descent. Am. J. Hum. Genet 97(3):404–18 26299365 10.1016/j.ajhg.2015.07.012 PMC4564943

[11] Browning SR, Browning BL, Daviglus ML, Durazo-Arvizu RA, Schneiderman N, 2018. Ancestry-specific recent effective population size in the Americas. PLOS Genet. 14(5):e1007385 29795556 10.1371/journal.pgen.1007385 PMC5967706

[12] Caye K, Deist TM, Martins H, Michel O, François O. 2016. TESS3: fast inference of spatial population structure and genome scans for selection. Mol. Ecol. Resour 16(2):540–48 26417651 10.1111/1755-0998.12471

[13] Charlesworth D. 2006. Balancing selection and its effects on sequences in nearby genome regions. PLOS Genet. 2(4):e64 16683038 10.1371/journal.pgen.0020064 PMC1449905

[14] Charlesworth B. 2012. The effects of deleterious mutations on evolution at linked sites. Genetics 190(1):5–22 22219506 10.1534/genetics.111.134288 PMC3249359

[15] Chen H, Patterson N, Reich D. 2010. Population differentiation as a test for selective sweeps. Genome Res. 20(3):393–402 20086244 10.1101/gr.100545.109 PMC2840981

[16] Cheng JY, Mailund T. 2015. Ancestral population genomics using coalescence hidden Markov models and heuristic optimisation algorithms. Comput. Biol. Chem 57:80–92 25819138 10.1016/j.compbiolchem.2015.02.001

[17] Cheng JY, Mailund T. 2020. Ancestral population genomics with Jocx, a coalescent hidden Markov model. In Statistical Population Genomics, ed. Dutheil JY, pp. 167–89. Berlin: Springer 10.1007/978-1-0716-0199-0_8 31975168

[18] Cheng X, DeGiorgio M. 2019. Detection of shared balancing selection in the absence of trans-species polymorphism. Mol. Biol. Evol 36(1):177–99 30380122 10.1093/molbev/msy202 PMC6530816

[19] Cheng X, DeGiorgio M. 2020. Flexible mixture model approaches that accommodate footprint size variability for robust detection of balancing selection. Mol. Biol. Evol 37(11):3267–91 32462188 10.1093/molbev/msaa134 PMC7820363

[20] Coop G, Witonsky D, Di Rienzo A, Pritchard JK. 2010. Using environmental correlations to identify loci underlying local adaptation. Genetics 185(4):1411–23 20516501 10.1534/genetics.110.114819 PMC2927766

[21] DeGiorgio M, Huber CD, Hubisz MJ, Hellmann I, Nielsen R. 2016. SweepFinder2: increased sensitivity, robustness and flexibility. Bioinformatics 32(12):1895–97 27153702 10.1093/bioinformatics/btw051

[22] DeGiorgio M, Lohmueller KE, Nielsen R. 2014. A model-based approach for identifying signatures of ancient balancing selection in genetic data. PLOS Genet. 10(8):e1004561 25144706 10.1371/journal.pgen.1004561 PMC4140648

[23] Diaz-Papkovich A, Anderson-Trocmé L, Ben-Eghan C, Gravel S. 2019. UMAP reveals cryptic population structure and phenotype heterogeneity in large genomic cohorts. PLOS Genet. 15(11):e1008432 31675358 10.1371/journal.pgen.1008432 PMC6853336

[24] Dilber E, Terhorst J. 2022. Robust detection of natural selection using a probabilistic model of tree imbalance. Genetics 220(3):iyac009 35100408 10.1093/genetics/iyac009 PMC8893258

[25] Elyashiv E, Sattath S, Hu TT, Strutsovsky A, McVicker G, 2016. A genomic map of the effects of linked selection in *Drosophila*. PLOS Genet. 12(8):e1006130 27536991 10.1371/journal.pgen.1006130 PMC4990265

[26] Engelhardt BE, Stephens M. 2010. Analysis of population structure: a unifying framework and novel methods based on sparse factor analysis. PLOS Genet. 6(9):e1001117 20862358 10.1371/journal.pgen.1001117 PMC2940725

[27] Ewens WJ. 2010. Mathematical Population Genetics, Vol. 1: Theoretical Introduction. Berlin: Springer. 2nd ed.

[28] Excoffier L, Marchi N, Marques DA, Matthey-Doret R, Gouy A, Sousa VC. 2021. *fastsimcoal2*: demographic inference under complex evolutionary scenarios. Bioinformatics 37(24):4882–85 34164653 10.1093/bioinformatics/btab468 PMC8665742

[29] Eyre-Walker A. 2010. Genetic architecture ofa complex trait and its implications for fitness and genome-wide association studies. PNAS 107:1752–56 20133822 10.1073/pnas.0906182107 PMC2868283

[30] Eyre-Walker A, Keightley PD. 2007. The distribution of fitness effects of new mutations. Nat. Rev. Genet 8(8):610–18 17637733 10.1038/nrg2146

[31] Fagundes NJR, Ray N, Beaumont M, Neuenschwander S, Salzano FM, 2007. Statistical evaluation of alternative models of human evolution. PNAS 104(45):17614–19 17978179 10.1073/pnas.0708280104 PMC2077041

[32] Fay JC, Wu CI. 2000. Hitchhiking under positive Darwinian selection. Genetics 155(3):1405–13 10880498 10.1093/genetics/155.3.1405 PMC1461156

[33] Ferrer-Admetlla A, Liang M, Korneliussen T, Nielsen R. 2014. On detecting incomplete soft or hard selective sweeps using haplotype structure. Mol. Biol. Evol 31(5):1275–91 24554778 10.1093/molbev/msu077 PMC3995338

[34] Field Y, Boyle EA, Telis N, Gao Z, Gaulton KJ, 2016. Detection of human adaptation during the past 2000 years. Science 354(6313):760–64 27738015 10.1126/science.aag0776 PMC5182071

[35] Flagel L, Brandvain Y, Schrider DR. 2019. The unreasonable effectiveness of convolutional neural networks in population genetic inference. Mol. Biol. Evol 36(2):220–38 30517664 10.1093/molbev/msy224 PMC6367976

[36] Foll M, Gaggiotti O. 2008. A genome-scan method to identify selected loci appropriate for both dominant and codominant markers: a Bayesian perspective. Genetics 180(2):977–93 18780740 10.1534/genetics.108.092221 PMC2567396

[37] Fu YX, Li WH. 1993. Statistical tests of neutrality of mutations. Genetics 133(3):693–709 8454210 10.1093/genetics/133.3.693 PMC1205353

[38] Galimberti M, Leuenberger C, Wolf B, Szilágyi SM, Foll M, Wegmann D. 2020. Detecting selection from linked sites using an F-model. Genetics 216(4):1205–15 33067324 10.1534/genetics.120.303780 PMC7768260

[39] Gao Z, Przeworski M, Sella G. 2015. Footprints of ancient-balanced polymorphisms in genetic variation data from closely related species. Evolution 69(2):431–46 25403856 10.1111/evo.12567 PMC4335603

[40] Garud NR, Messer PW, Buzbas EO, Petrov DA. 2015. Recent selective sweeps in North American *Drosophila melanogaster* show signatures of soft sweeps. PLOS Genet. 11(2):e1005004 25706129 10.1371/journal.pgen.1005004 PMC4338236

[41] Good BH, McDonald MJ, Barrick JE, Lenski RE, Desai MM. 2017. The dynamics of molecular evolution over 60,000 generations. Nature 551(7678):45–50 29045390 10.1038/nature24287 PMC5788700

[42] Gower G, Ragsdale AP, Bisschop G, Gutenkunst RN, Hartfield M, 2022. Demes: a standard format for demographic models. Genetics 222(3):iyac131 36173327 10.1093/genetics/iyac131 PMC9630982

[43] Gravel S. 2012. Population genetics models of local ancestry. Genetics 191(2):607–19 22491189 10.1534/genetics.112.139808 PMC3374321

[44] Gravel S, Henn BM, Gutenkunst RN, Indap AR, Marth GT, 2011. Demographic history and rare allele sharing among human populations. PNAS 108(29):11983–88 21730125 10.1073/pnas.1019276108 PMC3142009

[45] Gronau I, Hubisz MJ, Gulko B, Danko CG, Siepel A. 2011. Bayesian inference of ancient human demography from individual genome sequences. Nat. Genet 43(10):1031–34 21926973 10.1038/ng.937 PMC3245873

[46] Gutenkunst RN, Hernandez RD, Williamson SH, Bustamante CD. 2009. Inferring the joint demographic history of multiple populations from multidimensional SNP frequency data. PLOS Genet. 5(10):e1000695 19851460 10.1371/journal.pgen.1000695 PMC2760211

[47] Haak W, Lazaridis I, Patterson N, Rohland N, Mallick S, 2015. Massive migration from the steppe was a source for Indo-European languages in Europe. Nature 522(7555):207–11 25731166 10.1038/nature14317 PMC5048219

[48] Haller BC, Messer PW. 2022. SLiM 4: multispecies eco-evolutionary modeling. Am. Nat 201(5):E127–39 10.1086/723601 PMC10793872 37130229

[49] Harney É, Patterson N, Reich D, Wakeley J. 2021. Assessing the performance of qpAdm: a statistical tool for studying population admixture. Genetics 217(4):iyaa045 33772284 10.1093/genetics/iyaa045 PMC8049561

[50] Harris AM, DeGiorgio M. 2020. A likelihood approach for uncovering selective sweep signatures from haplotype data. Mol. Biol. Evol 37(10):3023–46 32392293 10.1093/molbev/msaa115 PMC7530616

[51] Harris AM, DeGiorgio M. 2020. Identifying and classifying shared selective sweeps from multilocus data. Genetics 215(1):143–71 32152048 10.1534/genetics.120.303137 PMC7198270

[52] Hein J, Schierup MH, Wiuf C. 2005. Gene Genealogies, Variation and Evolution: A Primer in Coalescent Theory. Oxford, UK: Oxford Univ. Press

[53] Hejase HA, Mo Z, Campagna L, Siepel A. 2022. A deep-learning approach for inference of selective sweeps from the ancestral recombination graph. Mol. Biol. Evol 39(1):msab332 34888675 10.1093/molbev/msab332 PMC8789311

[54] Hellenthal G, Busby GBJ, Band G, Wilson JF, Capelli C, 2014. A genetic atlas of human admixture history. Science 343(6172):747–51 24531965 10.1126/science.1243518 PMC4209567

[55] Hermisson J, Pennings PS. 2005. Soft sweeps: molecular population genetics of adaptation from standing genetic variation. Genetics 169(4):2335–52 15716498 10.1534/genetics.104.036947 PMC1449620

[56] Hernandez RD, Kelley JL, Elyashiv E, Melton SC, Auton A, 2011. Classic selective sweeps were rare in recent human evolution. Science 331(6019):920–24 21330547 10.1126/science.1198878 PMC3669691

[57] Huang YF, Gulko B, Siepel A. 2017. Fast, scalable prediction of deleterious noncoding variants from functional and population genomic data. Nat. Genet 49(4):618–24 28288115 10.1038/ng.3810 PMC5395419

[58] Hudson RR. 2002. Generating samples under a Wright-Fisher neutral model of genetic variation. Bioinformatics 18(2):337–38 11847089 10.1093/bioinformatics/18.2.337

[59] Hunter-Zinck H, Clark AG. 2015. Aberrant time to most recent common ancestor as a signature of natural selection. Mol. Biol. Evol 32(10):2784–97 26093129 10.1093/molbev/msv142 PMC4654765

[60] Johri P, Aquadro CF, Beaumont M, Charlesworth B, Excoffier L, 2022. Recommendations for improving statistical inference in population genomics. PLOS Biol. 20(5):e3001669 35639797 10.1371/journal.pbio.3001669 PMC9154105

[61] Johri P, Pfeifer SP, Jensen JD. 2023. Developing an evolutionary baseline model for humans: jointly inferring purifying selection with population history. Mol. Biol. Evol 40(5):msad100 37128989 10.1093/molbev/msad100 PMC10195113

[62] Johri P, Riall K, Becher H, Excoffier L, Charlesworth B, Jensen JD. 2021. The impact of purifying and background selection on the inference of population history: problems and prospects. Mol. Biol. Evol 38(7):2986–3003 33591322 10.1093/molbev/msab050 PMC8233493

[63] Jouganous J, Long W, Ragsdale AP, Gravel S. 2017. Inferring the joint demographic history of multiple populations: beyond the diffusion approximation. Genetics 206(3):1549–67 28495960 10.1534/genetics.117.200493 PMC5500150

[64] Kamm J, Terhorst J, Durbin R, Song YS. 2020. Efficiently inferring the demographic history of many populations with allele count data. J. Am. Stat. Assoc 115(531):1472–87 33012903 10.1080/01621459.2019.1635482 PMC7531012

[65] Kaplan NL, Darden T, Hudson RR. 1988. The coalescent process in models with selection. Genetics 120(3):819–29 3066685 10.1093/genetics/120.3.819 PMC1203559

[66] Kaplan NL, Hudson RR, Langley CH. 1989. The “hitchhiking effect” revisited. Genetics 123(4):887–99 2612899 10.1093/genetics/123.4.887 PMC1203897

[67] Kelleher J, Wong Y, Wohns AW, Fadil C, Albers PK, McVean G. 2019. Inferring whole-genome histories in large population datasets. Nat. Genet 51(9):1330–38 31477934 10.1038/s41588-019-0483-y PMC6726478

[68] Kern AD, Schrider DR. 2018. diploS/HIC: an updated approach to classifying selective sweeps. G3 8(6):1959–70 29626082 10.1534/g3.118.200262 PMC5982824

[69] Kim BY, Huber CD, Lohmueller KE. 2017. Inference of the distribution of selection coefficients for new nonsynonymous mutations using large samples. Genetics 206(1):345–61 28249985 10.1534/genetics.116.197145 PMC5419480

[70] Kim Y, Nielsen R. 2004. Linkage disequilibrium as a signature of selective sweeps. Genetics 167(3):1513–24 15280259 10.1534/genetics.103.025387 PMC1470945

[71] Kimura M. 1964. Diffusion models in population genetics. J. Appl. Probab 1(2):177–232

[72] Kingman JFC. 1982. The coalescent. Stoch. Process. Appl 13(3):235–48

[73] Kreiner JM, Latorre SM, Burbano HA, Stinchcombe JR, Otto SP, 2022. Rapid weed adaptation and range expansion in response to agriculture over the past two centuries. Science 378(6624):1079–85 36480621 10.1126/science.abo7293

[74] Kuhlwilm M, Han S, Sousa VC, Excoffier L, Marques-Bonet T. 2019. Ancient admixture from an extinct ape lineage into bonobos. Nat. Ecol. Evol 3(6):957–65 31036897 10.1038/s41559-019-0881-7

[75] Lachance J, Tishkoff SA. 2013. Population genomics of human adaptation. Annu. Rev. Ecol. Evol. Syst 44:123–43 25383060 10.1146/annurev-ecolsys-110512-135833 PMC4221232

[76] Lange JD, Pool JE. 2016. A haplotype method detects diverse scenarios of local adaptation from genomic sequence variation. Mol. Ecol 25(13):3081–100 27135633 10.1111/mec.13671 PMC4931985

[77] Lauterbur ME, Cavassim MIA, Gladstein AL, Gower G, Pope NS, 2023. Expanding the stdpopsim species catalog, and lessons learned for realistic genome simulations. eLife 12:e84874 10.7554/eLife.84874 PMC10328510 37342968

[78] Lawson DJ, Hellenthal G, Myers S, Falush D. 2012. Inference of population structure using dense haplotype data. PLOS Genet. 8(1):e1002453 22291602 10.1371/journal.pgen.1002453 PMC3266881

[79] Lazaridis I, Patterson N, Mittnik A, Renaud G, Mallick S, 2014. Ancient human genomes suggest three ancestral populations for present-day Europeans. Nature 513(7518):409–13 25230663 10.1038/nature13673 PMC4170574

[80] Lee S, Zou F, Wright FA. 2010. Convergence and prediction of principal component scores in high-dimensional settings. Ann. Stat 38(6):3605–29 21442047 10.1214/10-AOS821 PMC3062912

[81] Li H. 2011. A new test for detecting recent positive selection that is free from the confounding impacts of demography. Mol. Biol. Evol 28(1):365–75 20709734 10.1093/molbev/msq211

[82] Li H, Durbin R. 2011. Inference of human population history from individual whole-genome sequences. Nature 475(7357):493–96 21753753 10.1038/nature10231 PMC3154645

[83] Li W, Cerise JE, Yang Y, Han H. 2017. Application of t-SNE to human genetic data. J. Bioinform. Comput. Biol 15(4):1750017 28718343 10.1142/S0219720017500172

[84] Liu X, Fu YX. 2020. Stairway Plot 2: demographic history inference with folded SNP frequency spectra. Genome Biol. 21(1):280 33203475 10.1186/s13059-020-02196-9 PMC7670622

[85] Loh PR, Lipson M, Patterson N, Moorjani P, Pickrell JK, 2013. Inferring admixture histories of human populations using linkage disequilibrium. Genetics 193(4):1233–54 23410830 10.1534/genetics.112.147330 PMC3606100

[86] Luu K, Bazin E, Blum MG. 2017. *pcadapt*: an R package to perform genome scans for selection based on principal component analysis. Mol. Ecol. Resour 17(1):67–77 27601374 10.1111/1755-0998.12592

[87] Maier R, Flegontov P, Flegontova O, Işıldak U, Changmai P, 2023. On the limits of fitting complex models of population history to F-statistics. eLife 12:e85492 37057893 10.7554/eLife.85492 PMC10310323

[88] Marchi N, Schlichta F, Excoffier L. 2021. Demographic inference. Curr. Biol 31(6):R276–79 33756135 10.1016/j.cub.2021.01.053

[89] Marciniak S, Perry GH. 2017. Harnessing ancient genomes to study the history of human adaptation. Nat. Rev. Genet 18(11):659–74 28890534 10.1038/nrg.2017.65

[90] Marcus J, Ha W, Barber RF, Novembre J, Perry GH, 2021. Fast and flexible estimation of effective migration surfaces. eLife 10:e61927 34328078 10.7554/eLife.61927 PMC8324296

[91] McVean G. 2009. A genealogical interpretation of principal components analysis. PLOS Genet. 5(10):e1000686 19834557 10.1371/journal.pgen.1000686 PMC2757795

[92] McVicker G, Gordon D, Davis C, Green P. 2009. Widespread genomic signatures of natural selection in hominid evolution. PLOS Genet. 5(5):1000471 10.1371/journal.pgen.1000471 PMC2669884 19424416

[93] Moorjani P, Hellenthal G. 2023. Methods for assessing population relationships and history using genomic data. Annu. Rev. Genom. Hum. Genet 24:305–32 10.1146/annurev-genom-111422-025117 PMC11040641 37220313

[94] Moorjani P, Patterson N, Hirschhorn JN, Keinan A, Hao L, 2011. The history of African gene flow into Southern Europeans, Levantines, and Jews. PLOS Genet. 7(4):e1001373 21533020 10.1371/journal.pgen.1001373 PMC3080861

[95] Mughal MR, Koch H, Huang J, Chiaromonte F, DeGiorgio M. 2020. Learning the properties of adaptive regions with functional data analysis. PLOS Genet. 16(8):e1008896 32853200 10.1371/journal.pgen.1008896 PMC7480868

[96] Murphy DA, Elyashiv E, Amster G, Sella G, Nordborg M, Weigel D. 2022. Broad-scale variation in human genetic diversity levels is predicted by purifying selection on coding and non-coding elements. eLife 12:e76065 10.7554/eLife.76065 PMC10299832 36196994

[97] Navarro A, Barton NH. 2002. The effects of multilocus balancing selection on neutral variability. Genetics 161(2):849–63 12072479 10.1093/genetics/161.2.849 PMC1462137

[98] Nielsen R. 2005. Molecular signatures of natural selection. Annu. Rev. Genet 39:197–218 16285858 10.1146/annurev.genet.39.073003.112420

[99] Novembre J, Johnson T, Bryc K, Kutalik Z, Boyko AR, 2008. Genes mirror geography within Europe. Nature 456(7218):98–101 18758442 10.1038/nature07331 PMC2735096

[100] Orlando L, Allaby RG, Skoglund P, Sarkissian CD, Stockhammer PW, 2021. Ancient DNA analysis. Nat. Methods Rev. Primers 1:14

[101] Ortega-Del Vecchyo D, Lohmueller KE, Novembre J. 2022. Haplotype-based inference of the distribution of fitness effects. Genetics 220(4):iyac002 35100400 10.1093/genetics/iyac002 PMC8982047

[102] Palamara PF, Pe’er I. 2013. Inference of historical migration rates via haplotype sharing. Bioinformatics 29(13):i180–88 23812983 10.1093/bioinformatics/btt239 PMC3694674

[103] Palamara PF, Terhorst J, Song YS, Price AL. 2018. High-throughput inference of pairwise coalescence times identifies signals of selection and enriched disease heritability. Nat. Genet 50(9):1311–17 30104759 10.1038/s41588-018-0177-x PMC6145075

[104] Patterson N, Moorjani P, Luo Y, Mallick S, Rohland N, 2012. Ancient admixture in human history. Genetics 192(3):1065–93 22960212 10.1534/genetics.112.145037 PMC3522152

[105] Pavlidis P, Jensen JD, Stephan W. 2010. Searching for footprints of positive selection in whole-genome SNP data from nonequilibrium populations. Genetics 185(3):907–22 20407129 10.1534/genetics.110.116459 PMC2907208

[106] Pavlidis P, Živković D, Stamatakis A, Alachiotis N. 2013. SweeD: likelihood-based detection of selective sweeps in thousands of genomes. Mol. Biol. Evol 30(9):2224–34 23777627 10.1093/molbev/mst112 PMC3748355

[107] Peter BM. 2016. Admixture, population structure, and F-statistics. Genetics 202(4):1485–501 26857625 10.1534/genetics.115.183913 PMC4905545

[108] Peter BM, Huerta-Sanchez E, Nielsen R. 2012. Distinguishing between selective sweeps from standing variation and from a *de novo* mutation. PLOS Genet. 8(10):e1003011 23071458 10.1371/journal.pgen.1003011 PMC3469416

[109] Petkova D, Novembre J, Stephens M. 2016. Visualizing spatial population structure with estimated effective migration surfaces. Nat. Genet 48(1):94–100 26642242 10.1038/ng.3464 PMC4696895

[110] Pickrell JK, Pritchard JK. 2012. Inference of population splits and mixtures from genome-wide allele frequency data. PLOS Genet. 8(11):e1002967 23166502 10.1371/journal.pgen.1002967 PMC3499260

[111] Racimo F. 2016. Testing for ancient selection using cross-population allele frequency differentiation. Genetics 202(2):733–50 26596347 10.1534/genetics.115.178095 PMC4788246

[112] Ragsdale AP, Gravel S. 2019. Models of archaic admixture and recent history from two-locus statistics. PLOS Genet. 15(6):e1008204 31181058 10.1371/journal.pgen.1008204 PMC6586359

[113] Ragsdale AP, Gutenkunst RN. 2017. Inferring demographic history using two-locus statistics. Genetics 206(2):1037–48 28413158 10.1534/genetics.117.201251 PMC5499162

[114] Ralph P, Coop G. 2013. The geography of recent genetic ancestry across Europe. PLOS Biol. 11(5):e1001555 23667324 10.1371/journal.pbio.1001555 PMC3646727

[115] Rasmussen MD, Hubisz MJ, Gronau I, Siepel A. 2014. Genome-wide inference of ancestral recombination graphs. PLOS Genet. 10(5):e1004342 24831947 10.1371/journal.pgen.1004342 PMC4022496

[116] Reynolds J, Weir BS, Cockerham CC. 1983. Estimation of the coancestry coefficient: basis for a short-term genetic distance. Genetics 105(3):767–79 17246175 10.1093/genetics/105.3.767 PMC1202185

[117] Sabeti PC, Reich DE, Higgins JM, Levine HZ, Richter DJ, 2002. Detecting recent positive selection in the human genome from haplotype structure. Nature 419(6909):832–37 12397357 10.1038/nature01140

[118] Sabeti PC, Varilly P, Fry B, Lohmueller J, Hostetter E, 2007. Genome-wide detection and characterization of positive selection in human populations. Nature 449(7164):913–18 17943131 10.1038/nature06250 PMC2687721

[119] Sanjak JS, Sidorenko J, Robinson MR, Thornton KR, Visscher PM. 2018. Evidence of directional and stabilizing selection in contemporary humans. PNAS 115(1):151–56 29255044 10.1073/pnas.1707227114 PMC5776788

[120] Sawyer SA, Hartl DL. 1992. Population genetics of polymorphism and divergence. Genetics 132:1161–76 1459433 10.1093/genetics/132.4.1161 PMC1205236

[121] Schiffels S, Durbin R. 2014. Inferring human population size and separation history from multiple genome sequences. Nat. Genet 46(8):919–25 24952747 10.1038/ng.3015 PMC4116295

[122] Schlötterer C, Kofler R, Versace E, Tobler R, Franssen SU. 2015. Combining experimental evolution with next-generation sequencing: a powerful tool to study adaptation from standing genetic variation. Heredity 114(5):431–40 25269380 10.1038/hdy.2014.86 PMC4815507

[123] Sella G, Petrov DA, Przeworski M, Andolfatto P. 2009. Pervasive natural selection in the *Drosophila* genome? PLOS Genet. 5(6):e1000495 19503600 10.1371/journal.pgen.1000495 PMC2684638

[124] Sethuraman A, Hey J. 2016. IMa2p—parallel MCMC and inference of ancient demography under the isolation with migration (IM) model. Mol. Ecol. Resour 16(1):206–15 26059786 10.1111/1755-0998.12437 PMC4673045

[125] Shapiro B, Hofreiter M. 2014. A paleogenomic perspective on evolution and gene function: new insights from ancient DNA. Science 343(6169):1236573 24458647 10.1126/science.1236573

[126] Sheehan S, Harris K, Song YS. 2013. Estimating variable effective population sizes from multiple genomes: a sequentially Markov conditional sampling distribution approach. Genetics 194(3):647–62 23608192 10.1534/genetics.112.149096 PMC3697970

[127] Sheehan S, Song YS. 2016. Deep learning for population genetic inference. PLOS Comput. Biol 12(3):e1004845 27018908 10.1371/journal.pcbi.1004845 PMC4809617

[128] Siewert KM, Voight BF. 2020. BetaScan2: standardized statistics to detect balancing selection utilizing substitution data. Genome Biol. Evol 12(2):3873–77 32011695 10.1093/gbe/evaa013 PMC7058154

[129] Simons YB, Bullaughey K, Hudson RR, Sella G. 2018. A population genetic interpretation of GWAS findings for human quantitative traits. PLOS Biol. 16(3):e2002985 29547617 10.1371/journal.pbio.2002985 PMC5871013

[130] Smith CCR, Flaxman SM. 2020. Leveraging whole genome sequencing data for demographic inference with approximate Bayesian computation. Mol. Ecol. Resour 20(1):125–39 31512399 10.1111/1755-0998.13092

[131] Smith JM, Haigh J. 1974. The hitch-hiking effect of a favourable gene. Genet. Res 23(1):23–35 4407212

[132] Sousa da Mota B, Rubinacci S, Cruz Dávalos DI, Amorim CEG, Sikora M, 2023. Imputation of ancient human genomes. Nat. Commun 14:3660 37339987 10.1038/s41467-023-39202-0 PMC10282092

[133] Speidel L, Forest M, Shi S, Myers SR. 2019. A method for genome-wide genealogy estimation for thousands of samples. Nat. Genet 51(9):1321–29 31477933 10.1038/s41588-019-0484-x PMC7610517

[134] Spence JP, Steinrücken M, Terhorst J, Song YS. 2018. Inference of population history using coalescent HMMs: review and outlook. Curr. Opin. Genet. Dev 53:70–76 30056275 10.1016/j.gde.2018.07.002 PMC6296859

[135] Steinrücken M, Kamm J, Spence JP, Song YS. 2019. Inference of complex population histories using whole-genome sequences from multiple populations. PNAS 116(34):17115–20 31387977 10.1073/pnas.1905060116 PMC6708337

[136] Stern AJ, Wilton PR, Nielsen R. 2019. An approximate full-likelihood method for inferring selection and allele frequency trajectories from DNA sequence data. PLOS Genet. 15(9):e1008384 31518343 10.1371/journal.pgen.1008384 PMC6760815

[137] Sugden LA, Atkinson EG, Fischer AP, Rong S, Henn BM, Ramachandran S. 2018. Localization of adaptive variants in human genomes using averaged one-dependence estimation. Nat. Commun 9:703 29459739 10.1038/s41467-018-03100-7 PMC5818606

[138] Sul JH, Martin LS, Eskin E. 2018. Population structure in genetic studies: confounding factors and mixed models. PLOS Genet. 14(12):e1007309 30589851 10.1371/journal.pgen.1007309 PMC6307707

[139] Tajima F. 1989. Statistical method for testing the neutral mutation hypothesis by DNA polymorphism. Genetics 123(3):585–95 2513255 10.1093/genetics/123.3.585 PMC1203831

[140] Terhorst J, Kamm JA, Song YS. 2017. Robust and scalable inference of population history from hundreds of unphased whole genomes. Nat. Genet 49(2):303–9 28024154 10.1038/ng.3748 PMC5470542

[141] Torada L, Lorenzon L, Beddis A, Isildak U, Pattini L, 2019. ImaGene: a convolutional neural network to quantify natural selection from genomic data. BMC Bioinform. 20(9):337 10.1186/s12859-019-2927-x PMC6873651 31757205

[142] Vitti JJ, Grossman SR, Sabeti PC. 2013. Detecting natural selection in genomic data. Annu. Rev. Genet 47:97–120 24274750 10.1146/annurev-genet-111212-133526

[143] Voight BF, Kudaravalli S, Wen X, Pritchard JK. 2006. A map of recent positive selection in the human genome. PLOS Biol. 4(3):e72 16494531 10.1371/journal.pbio.0040072 PMC1382018

[144] Vy HMT, Kim Y. 2015. A composite-likelihood method for detecting incomplete selective sweep from population genomic data. Genetics 200(2):633–49 25911658 10.1534/genetics.115.175380 PMC4492385

[145] Wakeley J. 2008. Coalescent Theory: An Introduction. London: Freeman

[146] Wang K, Mathieson I, O’Connell J, Schiffels S. 2020. Tracking human population structure through time from whole genome sequences. PLOS Genet. 16(3):e1008552 32150539 10.1371/journal.pgen.1008552 PMC7082067

[147] Wang Z, Wang J, Kourakos M, Hoang N, Lee HH, 2021. Automatic inference of demographic parameters using generative adversarial networks. Mol. Ecol. Resour 21(8):2689–705 33745225 10.1111/1755-0998.13386 PMC8596911

[148] Willi Y, Kristensen TN, Sgrò CM, Weeks AR, Ørsted M, Hoffmann AA. 2022. Conservation genetics as a management tool: the five best-supported paradigms to assist the management of threatened species. PNAS 119(1):e2105076119 34930821 10.1073/pnas.2105076119 PMC8740573

[149] Wohns AW, Wong Y, Jeffery B, Akbari A, Mallick S, 2022. A unified genealogy of modern and ancient genomes. Science 375(6583):eabi8264 35201891 10.1126/science.abi8264 PMC10027547

[150] Wright S. 1943. Isolation by distance. Genetics 28(2):114–38 17247074 10.1093/genetics/28.2.114 PMC1209196

[151] Yang Z. 2007. PAML 4: phylogenetic analysis by maximum likelihood. Mol. Biol. Evol 24(8):1586–91 17483113 10.1093/molbev/msm088

[152] Yi X, Liang Y, Huerta-Sanchez E, Jin X, Cuo ZXP, 2010. Sequencing of 50 human exomes reveals adaptation to high altitude. Science 329(5987):75–78 20595611 10.1126/science.1190371 PMC3711608

[153] Zeng J, De Vlaming R, Wu Y, Robinson MR, Lloyd-Jones LR, 2018. Signatures of negative selection in the genetic architecture of human complex traits. Nat. Genet 50(5):746–53 29662166 10.1038/s41588-018-0101-4

